# Effect of Natural Zeolite (Clinoptilolite) on *in vitro* Biogenic Amine Production by Gram Positive and Gram Negative Pathogens

**DOI:** 10.3389/fmicb.2018.02585

**Published:** 2018-10-25

**Authors:** Fatih Özogul, Vida Šimat, Saadet Gokdogan, Joe M. Regenstein, Yesim Özogul

**Affiliations:** ^1^Department of Seafood Processing Technology, Faculty of Fisheries, Cukurova University, Adana, Turkey; ^2^Department of Marine Studies, University of Split, Split, Croatia; ^3^Department of Food Science, Cornell University, Ithaca, NY, United States

**Keywords:** clinoptilolite, tyramine, histamine, biogenic amines, food-borne pathogen

## Abstract

The effect of two levels of clinoptilolite (1 and 5%) on the production of biogenic amines (BA) and ammonia (AMN) by Gram positive (*Staphylococcus aureus, Enterococcus faecalis*, and *Listeria monocytogenes)* and Gram negative bacteria (*Aeromonas hydrophila, Klebsiella pneumoniae, Escherichia coli, Pseudomonas aeruginosa*, and *Salmonella* Parathypi A), in tyrosine decarboxylase broth (TDB) was studied. *A. hydrophila* and *E. coli* produced the highest amounts of amines which were 1223.06 and 2627.90 mg/l, respectively. All strains were able to decarboxylate tyrosine to tyramine (TYR) with *E. coli* being the highest (1657.19 mg/l). *A. hydrophila* formed >50 mg/l histamine (HIS) while the other strains produced none or very low concentrations (<4 mg/l). Among Gram-positive pathogens, *E. faecalis* was characterized as the main amine producer (478.23 mg/l). Although dependent on bacterial strain and level used, the natural zeolite clinoptilolite can be used to decrease BA and AMN production by bacterial strains that are of health concern.

**Practical Applications:** Uses of natural prodcuts for biogenic amines inhibition. Clinoptilolite was used to reduce the amounts of amines such as spermine, putrescine, and dopamine produced by pathogenic and spoilage bacteria.

## Introduction

Non-volatile organic nitrogenous compounds such as biogenic amines (BA) are found in a wide variety of foods, where they are formed by microbial decarboxylation of the precursor amino acids and their accumulation is related to bacterial spoilage thus they are considered undesirable compounds in food (Veciana-Nogués et al., 1997). The consumption of food containing high concentrations of BA has been associated with toxic effects and constitutes a potential health hazard and a food safety issue. The compounds mainly implicated in the direct toxic effect or interactions with some medical treatments are histamine (HIS) and tyramine (TYR).

The ability of bacteria to decarboxylate amino acids is highly variable as markedly different profiles of BA have been reported. This depends on various factors: the bacterial species, which are also strain-dependent, the availability of substrate amino acids, the presence of the intrinsic and extrinsic parameters of food that allow bacterial growth, the decarboxylase synthesis and decarboxylase activity (Özogul, 2011; Gokdogan et al., 2012). Species of somegenera such as *Staphylococcus, Enterococcus, Bacillus, Clostridium, Klebsiella, Escherihia, Proteus, Pseudomonas, Shigella, Photobacterium, Citrobacter* and the lactic acid bacteria have the capability to decarboxylate one or more amino acids producing high levels of BA in foods and different media (Özogul and Özogul, 2007; Gokdogan et al., 2012).

Gardini et al. (2016) and Doeun et al. (2017) reviewed factors affecting biogenic amine content in foods including hygienic of raw materials, microbial composition, fermentation condition, the use of starter culture, technological additives, effects of packaging, other non-thermal treatments, metabolizing BA by microorganisms, effects of pressure treatments on BA formation, and antimicrobial substances. The effective inhibitory effect of some food additives and spices (glycine, garlic, clove, cinnamon, and clinoptilolite) on BA production and growth of BA-forming bacteria had been reported (Uchida et al., 1992; Mah and Hwang, 2009; Gokdogan et al., 2012; Zhou et al., 2016).

Clinoptilolite (zeolites) is used in various applications such as a chemical sieve, a gas absorber, a feed additive, or food additive, an odor control agent and as a water filter for municipal and residential drinking water and aquariums due to its large amount of pore space, high resistance to extreme temperatures, and chemically neutral basic structure (Polat et al., 2004). Zeolites are microporous and crystalline aluminosilicate minerals that retain and slowly release cations. Their low coats and non-toxic and heat stable properties make them attractive carriers for antimicrobial metal ions such as Ag +. Cation exchange, especially silver exchange, is one of the properties of zeolites that give them prolonged antimicrobial properties (McDonnel and Pretzer, 2001; Top and Ülkü, 2004). This makes zeolites microbicide properties of great interest not only in food pathogen application, but over a wide range of applications against Gram-positive and Gram-negative bacteria (Narin et al., 2010; Johari et al., 2014). In histidine decarboxylase broth (HDB), the addition of clinoptilolite suppressed the BA production by Gram-negative FBP strains (Gokdogan et al., 2012) while *in vitro* studies showed its bactericidal effect against *P. aeruginosa, S. aureus*, and *E. coli* (Uchida et al., 1992). Additionally, the addition of a natural zeolite reduced BA in vacuum-packed sardines, especially HIS and TYR (Kuley et al., 2012). Since bacteria produce different amounts of BA in different decarboxylase broths, the aim of the study was to investigate BA and ammonia (AMN) formation by Gram-positive and Gram-negative food borne pathogens (FBP) in tyrosine decarboxylase broth (TDB) at two different levels (1 and 5%) of clinoptilolite.

## Materials and Methods

### Clinoptilolite

Clinoptilolite was provided by Enli Madencilik Sanayi ve Ticaret A.Ş., Izmir, Turkey.

### Foodborne Pathogens

The eight pathogens used were obtained from the American Type Culture Collection (Rockville, MD, United States). The bacteria were as follow: *Listeria monocytogenes* (ATCC7677), *Staphylococcus aureus* (ATCC29213), *Klebsiella pneumoniae* (ATCC700603), *Escherichia coli* (ATCC25922), *Enterococcus faecalis* (ATCC29212), and *Pseudomonas aeruginosa* (ATCC27853). *Aeromonas hydrophila* (NCIMB1135) was obtained from the National Collections of Industrial Food and Marine Bacteria (Aberdeen, United Kingdom) and *Salmonella* Parathypi A (NCTC13) was supplied by the National Collection of Type Cultures (London, United Kingdom).

### Culture Media and Extraction of the Bacterial Supernatant

The formation of TYR by all FBP strains was monitored using TDB according to Klausen and Huss (1987): One g peptone (Oxoid, Hamshire, England), 0.5 g Lab-Lemco powder (Oxoid, Hamshire, England), 2.5 g Sodium chloride, 4.01 g L-tyrosine HCl (Merck, Darmstadt, Germany), and 2.5 mg pyridoxal HCl (Merck, Darmstadt, Germany) were added into 500 ml distilled water. The initial pH of the broth was 6.6–6.8 and pH was adjusted depending on the optimum pH growth of pathogens with 1 M Sodium hydroxide or 0.1 M HCl using a WTW 315i pH Meter (Weilheim, Germany). Prior to use, the TDB was pipetted (10 ml bottles) and autoclaved for 15 min at 121°C.

Nutrient broth (Merck, Darmstadt, Germany) was used for the pathogen cultures. The bacterial strains were incubated for 2 or 3 days in their optimum growth temperature after which 0.5 ml of these pathogenic cultures were transferred to the TDB to perform the tyrosine decarboxylation. Two levels (1 and 5%) of clinoptilolite (Enli Madencilik Sanayi ve Ticaret A.Ş., Izmir, Turkey) were added.

For the extraction of supernatant from the FBP strains, 5 ml of the TDB that contains the pathogens were put in bottles with 2 ml trichloroacetic acid. Centrifugation was performed using a Hettich 32R centrifuge (Tuttlingen, Germany) at 3000 ×*g* for 10 min and then filtered through Whatman No. 1 filter paper (Maidenstone, United Kingdom). For the derivatization 4 ml of supernatant were taken from each sample.

### Chemical Reagents

L-tyrosine monohydrochloride (H8125) and all amine standards were bought from Sigma-Aldrich (Sigma-Aldrich, Munich, Germany). For amine and ammonia analyses acetonitrile and HPLC grade water were used.

### Preparation of Standard Amine Solution

For the preparation of the solution, amines were dissolved in 10 ml HPLC grade water, to reach a final concentration of 10 mg/ml solution for each free biogenic amine. The concentration was as followed: ammonium chloride (296.9 mg), putrescine dihydrochloride (182.9 mg), agmatine sulfate (175.4 mg), spermidine trihydrochloride (175.3 mg), spermine tetrahydrochloride (172.0 mg), cadaverine dihydrochloride (171.4 mg), HIS dihydrochloride (165.7 mg), trimethylamine hydrochloride (161.7 mg), 5-hydroxytryptamine (serotonin) (133.9 mg), 2-phenylethylamine hydrochloride (130.1 mg), Tyramine hydrochloride (126.7 mg), 3-hydroxytyramine hydrochloride (dopamine) (123.8 mg), and typtamine hydrochloride (122.8 mg).

### Derivatization

A stock solution was prepared by dissolving 2% benzoyl chloride (Sigma-Aldrich, Munich, Germany) in acetonitrile to improve the reaction with amines. For derivatization of standard amine solutions, 100 μl were taken (4 ml for extracted bacterial cultures) from each free BA standard solution (10 mg/ml). Sodium hydroxide (1 ml of 2 M) and 1 ml of 2% benzoyl chloride (dissolved in acetonitrile) were used and the solution was blended on a Vortex a ZX3 mixer (Welp Scientifica, Milano, Italy) for 1 min. The reaction mixture was left at room temperature (23^o^C) for 5 min and then centrifuged using a Hettich 32R centrifuge (Tuttlingen, Germany) at 3000 ×*g* for 10 min. Addition of 2 ml of saturated sodium chloride solution was used to stop the benzoylation and the solution was extracted with 2 ml of diethyl ether twice. The organic layer on top was relocated into a clean tube after blending. The organic layer was then removed by a stream of nitrogen until dryness. Prior to injection (10 μl aliquots) into HPLC, the residue was dissolved in 1 ml of acetonitrile.

### Analytical Method

Biogenic amines, ammonia, and trimethylamine were analyzed with Özogul (2004) method and measured as mg compound per liter broth. The rapid HPLC technique with a reversed phase column and a gradient elution program were used.

The HPLC instrument (Shimadzu, Kyoto, Japan) is equipped with a column oven (CTO-20AC), auto sampler (SIL 20AC), two binary gradient pumps (Shimadzu LC-10AT), SPD-M20A diode array detector, and a communication bus module (CBM-20A) with a valve unit FCV-11AL. A reverse-phase column, Spherisorb 5 Si C18 pH-St, 250 mm × 4.6 mm (Phenomenex, Macclesfield, Cheshire, United Kingdom) was used. Ammonia and TMA are measured using the same method with the other BA at the same injection.

BAs analysis were done using continuous gradient elution with acetonitrile (eluent A) and HPLC grade water (eluent B). The total separation time was 20 min and the injection volume was 10 μL. Detection wavelength was at 254 nm. A standard curve for ammonia and all amines in the range of 0–50 mg/mL was prepared. Correlation coefficient of peak area against amine standard concentrations for each compound was calculated after injecting five replicates of each standard solution of amine. The correlation coefficient (r2) in the curve was >0.99 for each benzoylated amine and ammonia.

### Data Analysis

The mean value and SD were calculated from four samples for each treatment. To determine the significance of differences one way ANOVA was used (*P* < 0.05). SPSS 15.0 for Windows (SPSS Inc., Chicago, IL, United States) was used to carry the statistics.

## Results and Discussion

### Biogenic Amine and Ammonia Production

The accumulation of AMN, BA, and trimethylamine (TMA) by Gram-negative and Gram-positive bacteria is shown in Table 1. All Gram-negative strains formed significant (*P* < 0.05) amounts of AMN (155 to 338 mg/l). The Gram-negative strains *A. hydrophila* and *E. coli* produced the highest total amounts of amines in TDB (1220 and 2630 mg/l, respectively). These two strains also produced the highest concentrations of AMN, PUT, CAD, phenylethylamine (PHEN), SPM, and AGM, making them the most potential toxic strains. *A. hydrophila* formed >50 mg/l HIS while other tested strains produced none or very low concentrations (<4 mg/l). *E coli* produced significantly (*P* < 0.05) more TYR than the other Gram-negative bacteria. Among the Gram-negative bacteria, *K. pneumonia* and *S. paratyphi* A were characterized as the lowest HIS and TYR producers. SPD, SER, PHEN, and DOP were generally produced in low to medium levels, with the exception of DOP production by *S. paratyphi* A and *A. hydrophila* that was somewhat higher. Tested FBP strains produced significant (*P* < 0.05) amount of SPM and AGM as well. Sixteen isolates of genus *Pseudomonas* were unable to generate BA according to some reports (Buňková et al., 2010). On the other hand, *Pseudomonas* spp. from Portuguese vacuum-packed cold-smoked fish was determined as good HIS producers (da Silva et al., 2002). Özogul and Özogul (2007) reported that different strains of *K. pneumoniae* accumulated different amounts of HIS, thus *K. pneumoniae* strain 673 formed >3400 mg/l HIS from histidine, whilst *E. faecalis, K. pneumoniae* (8152), and *K. pneumoniae* (2122) produced <10 mg/l HIS. In regard to the production of PUT and CAD, tested strains formed low to medium levels in TDB (Table 1). *Enterobacteriaceae* and *Pseudomonas* spp. are considered the main producers of PUT in various types of food, from fermented to chilled fish, meat, and cheese products (Wunderlichová, 2014).

**Table 1:** Ammonia and biogenic amines (BA) production by Gram-negative (G^-^) and Gram-positive (G ^+^) foodborne pathogens in tyrosine decarboxylase broth (TDB) (mg/l).

Pathogens		AMN	PUT	CAD	SPD	TRPT	PHEN	SPM	HIS	SER	TYR	TMA	DOP	AGM	Total BA production
*G^-^*	*A. hydrophila*	311.69^x^	70.87	29.33	5.74	2.05	16.21	211.51	57.19	15.71	566.56	2.25	78.38	167.26	1223.06
	(NCIMB1135)	(7.64)^ya^	(0.43)^a^	(2.29)^a^	(0.33)^a^	(0.07)^a^	(0.14)^a^	(4.93)^a^	(2.33)^a^	(0.32)^a^	(37.67)^a^	(0.18)^a^	(3.07)^a^	(15.57)^a^	(2.71)^a^
	*K. pneumoniae*	139.84	42.31	5.70	6.95	0.00	2.87	104.59	0.17	1.92	11.64	0.05	29.51	46.12	251.83
	(ATCC700603)	(4.16)^b^	(0.43)^b^	(0.28)^b^	(0.22)^b^	(0.00)^b^	(0.05)^b^	(0.49)^b^	(0.01)^b^	(0.10)^b^	(0.05)^b^	(0.00)^b^	(0.43)^b^	(0.85)^b^	(0.26)^b^
	*E. coli*	338.09	53.04	74.00	5.53	0.22	25.95	645.51	3.52	7.99	1657.19	0.23	8.82	146.12	2627.90
	(ATCC25922)	(12.89)^c^	(0.09)^c^	(0.58)^c^	(0.32)^a^	(0.00)^c^	(0.53)^c^	(46.33)^c^	(0.01)^c^	(0.61)^c^	(8.23)^c^	(0.01)^cd^	(0.19)^cd^	(2.57)^c^	(4.66)^c^
	*P. aeruginosa*	294.95	31.24	8.85	3.28	0.66	6.59	46.91	0.19	3.74	94.69	0.38	11.42	67.60	274.89
	(ATCC27853)	(18.32)^a^	(1.05)^d^	(0.21)^d^	(0.12)^c^	(0.01)^d^	(0.49)^d^	(0.48)^de^	(0.01)^b^	(0.19)^d^	(0.49)^d^	(0.03)^df^	(0.31)^c^	(0.92)^d^	(0.35)^b^
	*S.* Paratyphi A	154.61	8.34	6.67	0.00	0.00	4.74	24.83	0.00	1.01	21.03	0.45	99.40	23.16	189.63
	(NCTC13)	(7.49)^b^	(0.79)^e^	(0.03)^e^	(0.00)^d^	(0.00)^b^	(0.03)^e^	(1.04)^e^	(0.00)^b^	(0.01)^e^	(0.19)^b^	(0.04)^e^	(0.42)^d^	(0.50)^e^	(0.26)^d^
*G ^+^*	*S. aureus*	48.63	3.01	1.30	0.00	0.00	1.34	10.21	0.00	0.22	34.77	0.88	0.72	3.86	56.31
	(ATCC29213)	(0.92)^d^	(0.08)^f^	(0.00)^b^	(0.00)^d^	(0.00)^b^	(0.01)^f^	(0.06)^e^	(0.00)^b^	(0.00)^f^	(1.11)^b^	(0.03)^f^	(0.02)^e^	(0.18)^f^	(0.03)^e^
	*E. faecalis*	151.05	22.26	14.02	6.27	0.22	5.95	75.07	0.15	2.21	274.79	0.06	38.03	39.20	478.23
	(ATCC29212)	(4.79)^b^	(0.75)^g^	(0.68)^f^	(0.10)^e^	(0.02)^c^	(0.27)^d^	(3.37)^bd^	(0.01)^b^	(0.01)^b^	(0.19)^e^	(0.00)^b^	(1.20)^b^	(1.63)^b^	(0.73)^f^
	*L. monocytogenes*	143.24	7.42	1.65	0.00	0.68	2.99	29.19	0.12	2.43	37.33	0.10	31.60	8.99	122.50
	(ATCC7677)	(8.62)^b^	(0.31)^f^	(0.02)^e^	(0.00)^d^	(0.04)^d^	(0.00)^b^	(0.01)^e^	(0.02)^b^	(0.04)^b^	(0.41)^b^	(0.01)^bc^	(10.53)^b^	(0.33)^e^	(1.03)^g^

*AMN, ammonia; PUT, putrescine; CAD, cadaverine; HIS, histamine; SPD, spermidine; TRP, tryptamine; PHEN, 2-Phenylethylamine; SPN, spermine; SER, serotonin; TYR, tyramine; TMA, trimethylamine; DOP, Dopamine. AGM, agmatine. ^*x*^Mean value (*n* = 4), ^*y*^Standard deviation. Different uppercase letters^(*a*-*f*)^ in a column indicate significant differences (*P* < 0.05) among pathogens. *Listeria monocytogenes, Staphylococcus aureus, Klebsiella pneumoniae, Escherichia coli*, *Enterococcus faecalis*, and *Pseudomonas aeruginosa*. *Aeromonas hydrophila* and *Salmonella* Parathypi A*.

The formation of AMN and BA by foodborne pathogens in HDB, TDB, and lysine decarboxylase broth (LDB) was previously reported (Kuley and Özogul, 2011; Gokdogan et al., 2012; Özogul et al., 2016). In HDB and LDB five Gram-negative (*E. coli, K. pneumoniae, A. hydrophila, S.* Paratyphi A, and *P. aeruginosa*) and three Gram-positive FBP (*S. aureus, E. faecalis*, and *L. monocytogenes*) were able to decarboxylate more than one amino acid and produce not only HIS and CAD but also other amines.

In this work, Gram-positive pathogens formed lower concentrations of BA than Gram-negative bacteria and among them *E. faecalis* was demonstrated as the main amine producer (478.23 mg/l) (Table 1). It is interesting to point out the differences of our results to other obtained for AMN and BA production by Gram-positive bacteria in HDB and LDB. In HDB, Gokdogan et al. (2012) found *E. faecalis* to be the main producer of AMN (125.49 mg/l) and all investigated BA (production levels were <20 mg/l). In LDB, *S. aureus* produced the highest amounts of AMN (222.99 mg/l) and most BA (PUT; CAD, PHEN, HIS, and DOP) while *E. faecalis* produced high levels of SER, TYR, and AGM (Özogul et al., 2016). Interestingly, in HDB *E. faecalis* and *S. aureus* produced very low levels of HIS (<0.50 mg/l), CAD (<4 mg/l), and TYR (<5 mg/l) (Gokdogan et al., 2012) while in LDB the production levels were higher for all three BA, thus *E. faecalis* produced 65.24 mg/l of TYR while *S. aureus* produced 124.75 mg/l of CAD and 40.44 mg/l of HIS (Özogul et al., 2016). *L. monocytogenes* was characterized as the lowest AMN and BA Gram-positive producer in HDB (Gokdogan et al., 2012), TDB (this study) and LDB (Özogul et al., 2016). Beside AMN production (52.29 mg/l in HDB, 111.31 mg/l in LDB, and 143.24 mg/l in TDB), moderate levels of DOP (44.55 mg/l) formation in LDB (Özogul et al., 2016) and around 30 mg/l of TYR, SPM, and DOP production in TDB *L. monocytogenes* showed no ability to produce significant levels of BA.

Recently, there have been studies confirming enterococci species as tyramine- and putrescine-producer bacteria (Ladero et al., 2012; Marcobal et al., 2012). It was reported by Özogul and Özogul, 2007 that TYR was formed in highest concentration (526 mg/L) by *E. faecalis*. Various *E. faecalis* strains are able to yield TYR from 601 to 4986 μg/mL (Bover-Cid and Holzapfel, 1999; Connil et al., 2002). The strains of *E. faecalis* are very frequently found to be putrescine-producers (Llácer et al., 2007; Ladero et al., 2012), additionally our result confirm its ability to form significant concentrations of all tested BA in TDB. However, in HDB *E. faecalis* produced significantly lower concentrations of BA than in TDB, among them TYR, and PUT were 4.06 mg/l and 12.43 mg/l (Gokdogan et al., 2012). Kuley and Özogul (2011) reported accumulation of total amine formation as 330 mg/l for *A. hydrophila* and 884 mg/l for *E. faecalis* in TDB, and also *S.* Paratyphi A was characterized as the main amine former (4601 mg/l). In this study, *A. hydrophila* was observed as main AMN and BA producer in TDB, and *E. faecalis* as main Gram-positive producer, while total amine production by *S.* Paratyphi A was moderate, but lower than other bacterial strains (189.63 mg/l). In this study, *S.* Paratyphi A and *S. aureus* showed no ability to produce SPD, TRPT, or HIS. TMA was produced at low levels (0.05–2.25 mg/l) with *A. hydrophila* being the highest producer.

Gram-negative and positive FBPs showed the ability to produce significant amounts of TYR and other BA, as well as AMN and TMA in TDB. The most significant and prolific producer of TYR was Gram-negative strain *E. coli* (>1000 mg/l), followed by *A. hydrophila* (>500 mg/l) and Gram-positive *E. faecalis* (>205 mg/l). In TDB, these three strains produced the highest concentrations of other BA and AMN.

### The Influence of Natural Zeolite on AMN and BA Production by Gram-Positive and Gram-Negative FBP in TDB

**FIGURE 1:**
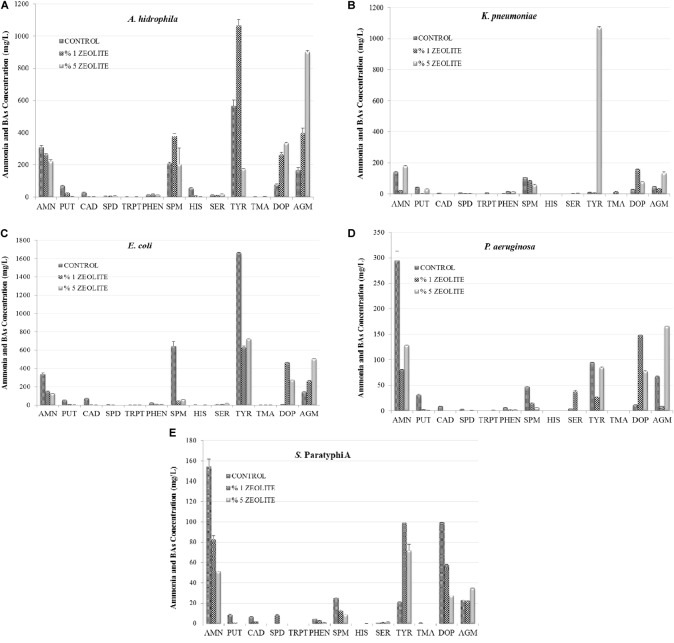
The effect of zeolite addition on amine formation (AMN, ammonia; PUT, putrescine; CAD, cadaverine; HIS, histamine; SPD, spermidine; TRP, tryptamine; PHEN, 2-Phenylethylamine; SPN, spermine; SER, serotonin; TYR, tyramine; TMA, trimethylamine; DOP, Dopamine; AGM, agmatine) by Gram-negative foodborne pathogens *A. hydrophila* **(A)**, *K. pneumoniae* **(B)**, *E. coli* **(C)**, *P. aeruginosa* **(D)**, *S.* Paratyphi A **(E)** in tyrosine decarboxylase broth.

The effect of zeolite on the studied compounds by Gram-negative FBP in TDB are shown in Figure 1. The 1% zeolite significantly reduced ANM production by all Gram-negative FBP, which were further reduced at 5% with the exception of *P. aeruginosa*. Generally, the effect of zeolite was dependent on bacterial strains and zeolite level since there was either stimulation or inhibition effect on the BAs production. For instance, 1% zeolite reduced TYR production by *E. coli* and *P. aeruginosa*, but 5% zeolite increased it. DOP and AGM production was stimulated at both 1 and 5% zeolite.

**FIGURE 2:**
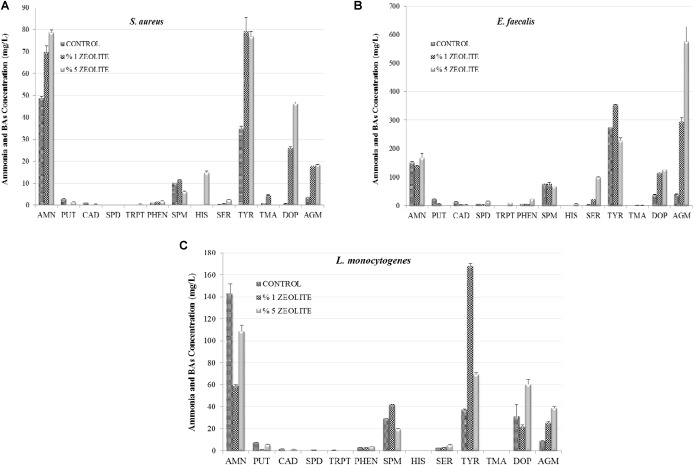
The influence of addition of zeolite on amine formation (AMN, ammonia; PUT, putrescine; CAD, cadaverine; HIS, histamine; SPD, spermidine; TRP, tryptamine; PHEN, 2-Phenylethylamine; SPN, spermine; SER, serotonin; TYR, tyramine; TMA, trimethylamine; DOP, Dopamine; AGM, agmatine) by Gram-positive foodborne pathogens *S. aureus* **(A)**, *E. faecalis* **(B)**, *L. momocytogenes* **(C)** in tyrosine decarboxylase broth.

Figure 2 shows the influence of zeolite on AMN and BA production by Gram-positive FBP. The 1% zeolite lowered AMN production by *L. monocytogenes* and *E. faecalis* significantly (*P* < 0.05). The elimination of AMN by natural zeolites has been well investigated, especially in wastewater treatment where natural zeolite was found very efficient (for 50 mg/l AMN 99.74% removal) (Wang and Peng, 2010; Zabochnicka-Świątek and Maliñska, 2010; Widiastuti et al., 2011). Additionally, the effect of natural clinoptilolite on AMN and other BA formation by FBP was studied in HDB and LDB (Gokdogan et al., 2012; Özogul et al., 2016). As in this work, in both studies the influence of zeolite was linked to the bacterial strains and to the zeolite concentrations. AMN production was higher in HDB with 1% zeolite while the zeolite addition showed a suppressive effect on HIS and especially TYR formations by Gram-negative FBP that were more sensitive compared to Gram-positive bacteria. Gokdogan et al. (2012) found that zeolite eliminated HIS accumulation by *S.* paratyphi A, *E. coli*, and *P. aeruginosa* (*P* < 0.05), but stimulated HIS formation by *A. hydrophila* and *K. pneumonia*. The addition of zeolite at 1% level increased TYR generation by *Aeromonas* spp. and *Escherichia* spp. and it was also determined that the 5% zeolite stimulated TYR production by *Pseudomonas* spp. and *K*. *pneumonia*. Özogul et al. (2016) also found that *E. coli* was able to decrease the AMN content as 132.35 mg/l (1% zeolite) and 121.21 mg/l (5% zeolite) were determined in LDB. This effect was observed for *S.* Paratyphi A but only at 5% level. Regarding the other bacteria important inhibitions of 5% zeolite was detected on AMN formation by *K. pneumoniae* (from 126.15 to 91.92 mg/l), *P. aeruginosa* (from 120.32 to 88 mg/l), and *A. hydrophila* (from 239.93 to 126.78 mg/l). On the other hand the adding zeolite at 1% elevated the AMN generation by *S.* Paratyphi from 83.01 to 87.05 mg/l (Özogul et al., 2016).

In this work the inhibitor effect of 1% zeolite was observed in PUT and CAD accumulation by all three tested Gram-positive FBP. Although HIS production by *S. aureus* and *E. faecalis* was very low or none in control group, there was a little stimulation effect of 5% zeolite addition in TDB. *L. monocytogenes* showed no ability to produce significant HIS in these conditions (0.12 mg/l), but the formation of SPM, TYR, and AGM was stimulated in presence of 1% zeolite (41.98, 168.23, and 25.77 mg/l, respectively). Addition of zeolite also simulated TYR, SER, DOP and AGM accumulations by Gram-positive FBP in TDB. The influence of zeolite on BA production by FBP in HDB has also shown dependence on pathogenic strains and zeolite concentrations (Gokdogan et al., 2012). Zeolite also showed to be suitable in food application; in a dynamic system such as fish. Its application had a direct effect on microbial growth in vacuum packed sardine filets and resulted in significant reduction of AMN and BA production during storage and at the same time its application improved sensory quality of the sardine in terms of off-odor removal (Kuley et al., 2012).

## Conclusion

This study showed that *A. hydrophila* and *E. coli* produced the highest amounts of amines which were 1220 and 2630 mg/l, respectively. All strains were able to decarboxylate tyrosine to TYR, especially *E. coli* (>1600 mg/l). Among the bacteria, *A. hydrophila* produced >50 mg/l HIS whereas the other strains formed none or very low levels of HIS (<4 mg/l). Gram-negative bacterial strains produced higher concentrations of AMN and BA in TDB, and zeolite addition had more apparent effect, especially for reducing AMN, PUT, CAD, HIS, and TYR production. In Gram-positive bacterial strains, the zeolite showed more stimulating effect on TYR production, but reduced CAD and PUT formation in three tested bacterial strains. In conclusion, zeolite can be used as a natural additive to prevent the production of some BAs such as CAD and PUT produced by Gram-negative FBPs.

## Author Contributions

FÖ and VŠ wrote the final version of the article. YÖ and SG performed all experiments, such as preparation of culture media and extraction of supernatant from pathogens, derivatization of biogenic amines. FÖ performed HPLC analyses of biogenic amines. JR edited the article.

## Conflict of Interest Statement

The authors declare that the research was conducted in the absence of any commercial or financial relationships that could be construed as a potential conflict of interest.
